# What Is Resilience and How Can It Be Nurtured? A Systematic Review of Empirical Literature on Organizational Resilience

**DOI:** 10.15171/ijhpm.2018.06

**Published:** 2018-02-06

**Authors:** Edwine Barasa, Rahab Mbau, Lucy Gilson

**Affiliations:** ^1^Health Economics Research Unit, KEMRI Wellcome Trust Research Programme, Nairobi, Kenya.; ^2^Nuffield Department of Medicine, University of Oxford, Oxford, UK.; ^3^School of Public Health and Family Medicine, University of Cape Town, Cape Town, South Africa.; ^4^Department of Global Health and Development, London School of Hygiene and Tropical Medicine, London, UK.

**Keywords:** Health System Resilience, Complex Adaptive Systems, Everyday Resilience, Health System Shocks

## Abstract

**Background:** Recent health system shocks such as the Ebola outbreak of 2014–2016 and the global financial crisis of 2008 have generated global health interest in the concept of resilience. The concept is however not new, and has been applied to other sectors for a longer period of time. We conducted a review of empirical literature from both the health and other sectors to synthesize evidence on organizational resilience.

**Methods:** We systematically searched for literature in PubMed, Econlit, EBSCOHOST databases, google, and Google Scholar and manually searched the reference lists of selected papers. We identified 34 papers that met our inclusion criteria. We analysed data from the selected papers by thematic review.

**Results:** Resilience was generally taken to mean a system’s ability to continue to meet its objectives in the face of challenges. The concepts of resilience that were used in the selected papers emphasized not just a system’s capacity to withstand shocks, but also to adapt and transform. The resilience of organizations was influenced by the following factors: Material resources, preparedness and planning, information management, collateral pathways and redundancy, governance processes, leadership practices, organizational culture, human capital, social networks and collaboration.

**Conclusion:** A common theme across the selected papers is the recognition of resilience as an emergent property of complex adaptive systems. Resilience is both a function of planning for and preparing for future crisis (planned resilience), and adapting to chronic stresses and acute shocks (adaptive resilience). Beyond resilience to acute shocks, the resilience of health systems to routine and chronic stress (everyday resilience) is also key. Health system software is as, if not more important, as its hardware in nurturing health system resilience.

## Background

Health systems globally have experienced major crisis and disruptive shocks over the past decade. This includes the 2008 global economic crisis^1^ and the 2014-2016 Ebola outbreak.^2^ These and previous shocks have catalysed the increasing attention to the concept of resilience in global health discourse.^2-4^ Building the resilience of health systems, it has been argued, will reduce their vulnerability to crisis, by ensuring that they are better prepared and effectively respond, and that there is maintenance or minimal disruption of the delivery of core healthcare services.^2,3,5,6^

However, despite its relatively recent entrance into the global health debates, the concept has been applied in other sectors for a longer period of time. The concept originated from the physical sciences, where it refers to a physical system’s capacity to return to its original form after a disturbance.^7^ The concept was subsequently applied to the ecological sciences where it was used to refer to an ecosystem’s ability to absorb shocks while maintaining function.^8,9^ This early conceptualization of resilience as a system’s ability to bounce back from a disturbance has been called *engineering resilience* as it was based on a “machine” view of systems, with simple cause and effect dynamics. However, subsequent applications of resilience, especially to social systems, recognized the complex adaptive nature of systems. This recognition prompted a view of resilience as involving the adaptation and transformation of systems though the emergence of new structures such as policies, processes and organizational culture that enable organizations to continue to perform their functions in the face of challenges.^10,11^

Despite the growing interest in the concept of resilience, there is scarce evidence on how to generate or strengthen resilience in health systems or in other sectors. The resilience literature is predominantly conceptual, focusing on concepts and principles.^12^ Yet understanding what makes systems resilient in the real world is critical to thinking about developing strategies for strengthening their resilience. We reviewed empirical literature with the aim of exploring how resilience was conceptualized, identifying the factors that influence organizational resilience and how they could be nurtured. Organizational resilience has been defined as ‘the maintenance of positive adjustment under challenging conditions such that the organization emerges from those conditions strengthened and more resourceful.’^13^ Because empirical literature on resilience is scarce, and because the health sector has a very short history of engaging with the concept, we deliberately broadened our review to include literature from both the health and other sectors. This is, to our knowledge, the first review of empirical literature on resilience undertaken to support health systems thinking, and it makes the following contributions. First, the evidence synthesized enriches debates on health system resilience by drawing on experiences from other sectors, so allowing for cross-pollination of ideas. Second, the evidence on factors that influence organizational resilience can inform the development of interventions for strengthening health system resilience, and developing frameworks for monitoring the impact of these interventions.

## Methods

### Literature Search

We searched literature in December 2016 in PubMed, Econlit, EBSCOHOST databases, google, and Google scholar. We used the following keywords to search for literature: ‘Resilience’ and ‘organization*’ or ‘organization’ or ‘institution’ or ‘system.’ We manually searched the reference lists of selected papers for relevant papers. We did not use any time restrictions in our search but included studies up to the time of the literature search (December 31, 2016). We used the following inclusion criteria to select papers to be included in the review: (1) papers published in the English language, (2) papers that reported empirical research on organizational resilience rather than theoretical/conceptual papers. Empirical research is based on observed and measured phenomena and derives knowledge from actual experience rather than from theory or belief, and (3) papers that reported studies that focused on organizational resilience, rather than the resilience of individuals. In this step, we first screened study abstracts and then obtained full-text formats for studies deemed relevant. Two authors independently reviewed all abstracts and full-text formats.

The first step in the literature search resulted in a total of 24 984 papers. Of the 24 984 articles, 24 708 articles were excluded after a review of abstracts because they were not empirical papers, and/or focused on individual rather than organizational resilience. Eight more articles were excluded for being duplicates. An assessment of the full-text formats of the remaining 268 papers resulted in a further 234 exclusions. A total of 34 studies were finally included in the review (Table 1). Figure outlines the screening process of papers obtained through searches.

**Table 1:** Characteristics of Selected Papers

**Author**	**County**	**Sector/System**	**Challenges to the System**	**Study Objective**
Achour and Price, 2010^14^	UK	Health systems	Manmade and natural disasters	To examine the resilience strategies in the UK healthcare system in the face of manmade and natural disasters
Ager et al, 2015^15^	Nigeria	Health systems	Insecurity - the Boko Haram terrorist insurgence	To examine the resilience of the health system in Yobe state in Nigeria to the insecurity caused by the Boko Haram terrorist insurgence
Andrew et al, 2016^16^	Thailand	government agencies, small businesses and corporations, and non-governmental organizations	Natural disasters (floods)	To examine whether bonding and bridging in social networks enhances the resilience of organizations (government agencies, small businesses and corporations, and non-governmental organizations) to natural disasters (floods) in Thailand
Beerman, 2011^17^	Germany	Food industry	Climate change	To examine the resilience of the German food industry to climate change
Booher and Innes, 2010^18^	US	Water management	Chronic economic, environmental, and political challenges	To explore governance strategies that enhanced the resilience of the California water management system to everyday economic, environmental, and political challenges in the US
Burke et al, 2014^19^	Ireland	Health systems	Economic crisis	To examine the resilience of the health system to the global economic crisis in Ireland
Christopher and Peck, 2005^20^	UK	Multiple industries: distribution of automotive spares, transport services, food retailing, pharmaceutical, oil and petrochemicals, electronics, packaging, private and public sector organizations	Acute shocks (natural disasters, industrial disputes, terrorism)	To examine the resilience of organizations in the transport sector to a range of acute shocks that include natural disasters, industrial disputes, and terrorism
Felland et al, 2003^21^	US	Health systems	Strained public budgets, and the introduction of laws that threatened to reduce the funding streams of safety net programmes	To determine the resilience of local health care safety nets in the US to economic challenges
Sawalha, 2015^22^	Jordan	Insurance companies	Everyday challenges (loss of customers, competition, political instability, financial losses)	To examine the resilience of general insurance companies in Jordan to everyday challenges (loss of customers, competition, political instability, financial losses)
Hassall et al, 2014^23^	Australia	Multiple sector: healthcare, oil, services and consulting, manufacturing, gas and refining, transport and logistics	Unspecified acute shocks	To examine the perspective of industry practitioners from multiple sectors (healthcare, oil, services and consulting, manufacturing, gas and refining, transport and logistics) on organizational resilience to unspecified acute shocks in Australia
Herrfahrdt-Paehle and Pahl-Wosrt, 2012^24^	South Africa and Uzbekistan	Socio - Ecological systems	Environmental changes	To examine the resilience of socio-ecological systems in South Africa and Uzbekistan to environmental changes
Heese et al, 2014^25^	Austria	Aviation	Unspecified crisis	To develop and validate a tool to assess the resilience of organizations in the aviation industry to unspecified acute crisis in Australia
Kachali et al, 2012^26^	New Zealand	Organizations in six industry sectors: trucking, fast-moving consumer goods, hospitality, information and communication technology, critical infrastructure, building suppliers	Earthquake	To examine the resilience of organizations drawn from 6 industry sectors (trucking, fast-moving consumer goods, hospitality, information and communication technology, critical infrastructure, building suppliers) to an earthquake in the Canterbury region of New Zealand
Lapao et al, 2015^27^	Lusophone African countries	Health systems	Disease outbreak - Ebola	To assess the resilience of Lusophone African countries to the Ebola disease outbreak
Lembani et al, 2014^28^	Ivory Coast	Health systems	Civil unrest following a disputed election	To examine the resilience of HIV service delivery to civil unrest in Ivory Coast
Lembani et al, 2015^29^	South Africa	Health systems	Everyday challenges including staff shortages, resource scarcity	To use a systems dynamic approach to examine the resilience of the health system to everyday challenges (including staff shortages and resource scarcity) in the Eastern Cape, OR Tambo district, South Africa
Mafabi et al, 2013^30^	Uganda	Parastatal organizations	Unspecified internal and external challenges	To examine the moderating effect of creative climate on knowledge management in influencing organizational resilience across 51 parastatal organizations in Uganda
McKenzie et al, 2015^31^	Nigeria	Health systems	Disease outbreak - Ebola	To examine the resilience of the Nigerian health system to the Ebola disease outbreak
McManus et al, 2007^32^	New Zealand	Public and private industries (local authority, private manufacturer, private contractor, education provider, public utility provider, private wholesale distributor, private retailer, private utility provider, private technology provider, private primary producer)	Acute manmade and natural shocks	To examine the resilience of 10 case study organizations (local authority, private manufacturer, private contractor, education provider, public utility provider, private wholesale distributor, private retailer, private utility provider, private technology provider, private primary producer) to acute manmade and natural shocks in New Zealand
McManus et al, 2008^33^	New Zealand	Public and private industries (local authority, private manufacturer, private contractor, education provider, public utility provider, private wholesale distributor, private retailer, private utility provider, private technology provider, private primary producer)	Acute manmade and natural shocks	To introduce a facilitated process that enhances resilience to manmade and natural disasters in 10 case study organizations (local authority, private manufacturer, private contractor, education provider, public utility provider, private wholesale distributor, private retailer, private utility provider, private technology provider, private primary producer) in New Zealand
Nilakant et al, 2013^34^	New Zealand	Four organizations in unspecified industries	Earthquake	To examine the resilience of 4 organizations in unspecified industries to the 2010-2011 earthquake in New Zealand
Nyikuri et al, 2015^35^	Kenya	Health systems	Rapid decentralization reforms	To examine the roles of primary healthcare facility managers in Kenya
Olsson et al, 2004^36^	Sweden and Canada	Socio - Ecological systems	Environmental changes	To examine how the resilience of social–ecological systems to environmental changes in Lake Racken in Western Sweden and estuaries of James bay in Canada
Oluwasoye and Ugonna, 2015^37^	Nigeria	Multinational Oil companies	Environmental risk and disasters (eg, gas flaring and oil spills)	To examine the resilience of multinational oil companies to environmental risk (eg, gas flaring and oil spills) in the Niger delta region of Nigeria
Andersson et al, 2012^38^	Sweden	Textile and clothing	Economic/financial crisis	To examine the resilience of Swedish textile industries to the global economic crisis of 2007-2011
Pal et al, 2014^39^	Sweden	Textile and clothing	Economic/financial crisis	To examine the resilience of textile-related small and medium enterprises to economic crisis in Sweden
Sandanda, 2009^40^	Zimbabwe	None specific organizational systems	Unspecified internal and external challenges	To investigate the influence of flexibility and business networks on organizational resilience to unspecified internal and external challenges in retail organizations in Harare, Zimbabwe
Sheffi and Rice, 2005^41^	US	Transport sector	Man-made and natural disasters	To examine the resilience of supply chain systems in the transport sector to manmade and natural disasters in the US
Seville et al, 2006^42^	New Zealand	Private and public, non-for-profit and for-profit and, small and large organizations within the roading network	Seismic, human and technological hazards	To examine the resilience of public and private organizations in the roading network to earthquakes, human and technological hazards in New Zealand
Seville et al, 2008^43^	New Zealand	Private and public, non-for-profit and for-profit and, small and large organizations within the roading network	Earthquakes, human and technological hazards	To develop strategies for improving the resilience of organizations to major crisis events such as earthquakes, human and technological hazards in New Zealand
Stephenson et al, 2010^44^	New Zealand	Multiple industry sectors (agriculture, communication, forestry and fishing, construction, education, cultural and recreational services, finance and insurance, health and community services, government administration and defense, manufacturing, personal and other services, retail trade and wholesale trade, property and business services)	Hazards in the natural, built and economic environment	To develop a web-based organizational resilience measurement and benchmarking tool which can provide organizations in the Auckland region of New Zealand with information to help make a business case for resilience
Thomas et al, 2013^1^	Ireland	Health systems	Economic crisis	To develop a framework for assessing the resilience of health systems to economic crisis in Ireland
Walker et al, 2014^45^	New Zealand	Infrastructure organizations (air travel, banking, telecommunications, water/waste services, roading)	Natural disasters such as earthquakes	To explore the relationship between work engagement and the resilience of 11 organizations (air travel, banking, telecommunications, water/waste services, roading) in Christchurch New Zealand following the Canterbury seismic events
Zhong et al, 2014^46^	China	Health systems	Disasters - manmade, natural and disease outbreaks	To explore the resilience of tertiary hospitals to manmade and natural disasters in Shandong province, China

**Figure:**
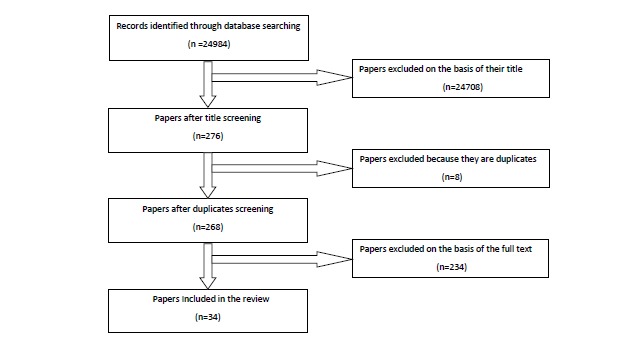
Screening Process of Papers Obtained Through Searches.

### Characteristics of Selected Studies

Table 1 outlines the characteristics of the selected papers. Even though we did not use any time restriction, the oldest publication that met our selection criteria was published in 1998 (less than 20 years old). This highlights the fact that empirical work on organizational resilience in health and other sectors is fairly recent. Of the 34 papers, 22 were focused on high income country experiences, while only 12 were based on low and middle income country experiences. Of the selected papers, 12 were based on health sector experiences, while 22 were based on other sector experiences. Two of the studies from outside the health sector focused on socio-ecological systems, while the rest focused on overall organizational resilience. Based on the selected papers, it appears that empirical work on organizational resilience has largely focused on identifying the characteristics that make systems resilient. This was either achieved by testing the association between a quantitative measure of organizational resilience and quantitative measures of resilience attributes derived from an a priori resilience framework (4 papers), or examining the experience of resilient systems in the face of challenges to identify enablers of resilience. For example, on the one hand, Sawalha^22^ applied an a priori framework to quantitatively assess the resilience of general insurance organisations in Jordan to multiple everyday challenges of competition, loss of customers, financial losses and political instability, while Hassall et al^23^ examined the perspectives of practitioners from multiple industries (healthcare, services and consulting, oil, gas and refining, manufacturing, transport and logistics) in Australia on organizational resilience to unspecified acute shocks. On the other hand, Achour and Price^14^ explored the healthcare resilience strategies of healthcare organizations in the United Kingdom to econoLembani mic challenges, while et al^28^ examined the mechanisms that influence the resilience of the Ivory Coast health system to disruptions caused by a civil war.

### Quality Appraisal

We used the Critical Appraisal Skills Programme (CASP) tool, which uses a check-list approach with screening questions, to assess the adequacy, trustworthiness and relevance of the evidence reported in the articles.^47,48^ The quality appraisal results are outlined in Table 2.

**Table 2:** Quality Appraisal Checklist

**Appraisal Criteria**	**Yes**	**Somewhat**	**No/Not Clear**
1. Was there a clear statement of the aims of the research?	34		
2. Is the methodology used for the study appropriate for addressing the research goal?	33		1
3. Was the research design appropriate to address the aims of the research? • Has the researcher justified the research design?	33		1
4. Is the recruitment strategy appropriate for the study aims? • Researcher explained how the study informants were selected and why these participants were the most appropriate? • Discussion around recruitment ie, why some people chose not to take part?	20	3	11
5. Was the data collected in a way that addressed the research issue? • If the setting for data collection was justified? • If it is clear how data were collected? • If the researcher has made the methods explicit?	24	3	7
6. Has the relationship between the researcher and the participants been adequately considered? • Researcher reflexivity and potential bias during the formulation of research questions or data collection?	2		32
7. Have ethical issues been taken into consideration? • Informed consent and confidentiality • Approval from ethics committee?	8		26
8. Was the data analysis sufficiently rigorous? • In-depth description of the analysis process? Clarity of the development of themes/categories • Are contradictory data taken into account?	20	8	6
9. Is there a clear statement of findings? • Explicit findings • Adequate discussion of evidence for and against the researcher arguments • Credibility of finds (triangulation, respondent validation, more than one analyst), findings are discussed in relation to the original research question)	32	2	
10. How valuable is the research? • Researcher discusses the contribution of the study to existing knowledge and understanding • If they identify new areas where research is possible? • If the researchers have discussed whether or how the findings can be transferred to other populations?	34		

A large number of selected papers scored poorly on explicating (1) the approach used to select study participants, (2) the relationship between the researchers and participants, and (3) measures taken to ensure the study adhered to research ethics standards. However, our observation what that this was largely because of difference in style and practice of science writing in health and other sectors. While papers from the health sector were structured in the common tradition of health/medical science (introduction, methods, results, discussion, conclusion) with each section providing significant detail, papers from other sectors were more heterogeneous in structure, and focused more on discussing findings, and less on explicating methods. We therefore interpreted the differences in quality score as difference in style and writing practice rather than necessarily difference in quality. As a result, we opted to include all the selected papers, as excluding some on the basis of the quality score would likely preferentially exclude papers from other sectors and we judged all papers as offering valuable insights for the review.

### Synthesis of Selected Papers

We conducted a thematic review of the selected papers.^49^ This entailed the following steps: (1) familiarizing with the data by reading through the selected papers, (2) generating a coding framework, (3) reading through the selected papers and coding the contents based on the coding framework (4) charting the coded data, and analyzing by constructing themes from these emergent ideas and concepts in an interpretive stage where findings from the selected papers were integrated into coherent themes. Coding was done in NVIVO version 10 software.

## Results

### Concept of Resilience

Across the selected papers, resilience was generally taken to mean a system’s ability to continue to perform and meet its objectives in the face of challenges. There is a general consensus around the notion that organizational resilience is achieved by a combination of absorbing the challenges faced, and changing by adapting and transforming so as to continue to thrive in the face of challenges. This is in contrast to the early notion of resilience as simply bouncing back from shock (resilience engineering). For instance, Herrfahrdt-Paehle and Pahl-Wostl,^24^ who examine the tension between continuity and change and how they affect the resilience of socio-ecological systems to environmental changes in South Africa and Uzbekistan, adopt Folke’s^9^ definition of resilience as a system’s capacity to absorb disturbances, while learning from them and reorganizing. While resilience engineering is grounded on a machine-like view of systems, with simple cause and effect relationships, organizational resilience that is conceptualized as the ability of an organization to absorb, adapt, and transform in the face of challenges is grounded on the view of systems as complex and adaptive.^50^

Walker et al,^45^ who explore the link between work engagement and the resilience of infrastructure organizations (air travel, banking, telecommunications, water/waste services, and roads) to earthquakes in New Zealand, distinguish between two dimensions of resilience; *planned resilience*, and *adaptive resilience*. Organizations exhibit planned resilience when they employ pre-existing plans to avoid or minimize the effect of a crisis. These include business continuity and risk management plans that outline pre-disaster activities required to keep organizations running during and after a period of disruptions such as natural (earth quakes, floods, disease outbreaks) and man-made disasters (terrorist attacks, fires).^45^ Adaptive resilience emerges during the post-disaster (natural and/or man-made) period as new capacities are developed by organizations by responding to emergent situations.^34^ Walker et al^45^ emphasize that while planned resilience is important, adaptive resilience is more influential since it is more sustainable and effective in the context of uncertainty about what the future could bring.

### Shocks and Challenges Faced by Systems and Organizations

A majority of the papers (9/12) that examined the resilience of health systems focused on acute, often catastrophic shocks to the system. Shocks are classified as acute if they are sudden in occurrence and transient in nature. These included disease outbreaks,^27,31^ insecurity,^15,29^ economic crisis,^1,19^ unspecified natural and/manmade disasters,^14,46^ and rapid policy reforms.^35^ Only 2 papers focused on the resilience of health systems to chronic, everyday challenges. Challenges are described as chronic if they persistent and recurrent over long periods of time. Lembani et al^29^ examined the resilience of the health system of South African provinces faced with chronic health system dysfunction and politicization. Felland et al^21^ examined the resilience of local healthcare safety nets to chronic economic pressures and budget cuts in the United States of America. Among the papers that examined resilience outside the health sector, there appears to be a focus on both acute shocks, such as earthquakes,^26,45^ environmental disasters,^37^ and other natural disasters,^16,20^ and multiple everyday challenges such as competition, financial difficulties, punitive laws, and climate change.^23,24^ It appears that the notion of resilience to chronic, everyday challenges has been embraced more by other sectors, compared to the health sector.

### Factors That Influence the Resilience of Organizations

#### Material Resources

The availability of resources is considered a key enabler of organizational resilience.^21,28,29,32,39^ When material resources are used strategically, organizations can overcome disruption. Financial resources are also considered necessary to mobilize other needed resources during crisis. For example, Pal et al^39^ observed that resource constraints, specifically material, ﬁnancial, and technological, impaired the resilience of small and middle enterprises to economic crisis in Sweden. McManus et al^32^ examined factors that influence the resilience of 10 case study organizations (private manufacturer, local authority, private contractor, public utility provider, private primary producer, education provider, private wholesale distributor, private utility provider, private retailer, private technology provider) to acute shocks (natural and manmade) in New Zealand and found that an organizations’ financial position was a key ingredient to its resilience.

#### Preparedness and Planning

Resilience to acute shocks, rather than everyday challenges, is enhanced by adequate planning.^17,26-28,32^ For example, hospitals in the United Kingdom developed and tested business continuity and risk management plans to ensure the continued functioning of core services throughout natural (such as floods) and man-made (such as terrorist attacks) disasters.^14^ McManus et al^32^ found that the degree to which organizations planned for continued supply of essential goods and services in times of a crisis contributed to the resilience of 10 case study organizations (local authority, private primary producer, private manufacturer, private contractor, public utility provider, private technology provider, private wholesale distributor, private retailer, education provider, private utility provider) in New Zealand. One of the strategies used by organizations to prepare for crises or disasters is by going through scenario exercises (pseudo-crisis situations). Lapao et al^27^ observes that health systems in Lusophone African countries, faced with the uncertain future of a disease outbreak, should, among others, prioritize the training of health professionals to seriously prepare them through scenario drills.

#### Information Management

Organizational resilience is widely identified as being dependent on how information is managed and used.^14-16,23,26,27,30,32,44^ For example, Ager et al^15^ found that the flow of information between the security services and state ministry of health strengthened health system resilience to the Boko Haram insurgency in Nigeria. Lapao et al^27^ found that a clear flow of information was necessary to allow a quick and correct response to the Ebola disease outbreak in Lusophone African countries. Information was seen as a key ingredient to how timely and adequately organizations adapted to challenges. According to Stephenson et al,^44^ knowledge management involves ensuring that strategies, organizational goals and achievements are effectively communicated across the organization. Further, organizations should proactively monitor what is happening in their environment. This could be achieved by activities such as evaluation of competitors, market research, and political and regulatory awareness.^44^ A key utility of effective information management and use was in enhancing the situation awareness of organizations. Situation awareness refers to an organization’s perception and understanding of its environment.^33^ Situation awareness is characterized by an increased understanding of the factors that trigger crisis, minimum operating requirements, availability of internal and external resources. Organizations can identify the early warning signals that precede a crisis by monitoring internal and external environments. Reflecting on the experience of the Ebola disease outbreak in Lusophone African countries, Lapao et al^27^ recommend the need for effective information and epidemiological surveillance systems that monitor and report on the status of the system and provide real time early warning of impending health threats.

#### Collateral Pathways and Redundancy

Ensuring that organizations have multiple, alternative courses of action also bestows resilience.^15,21,28,29,32,38,44,46^ According to Lembani et al^28^ collateral pathways refer to the availability of alternative routes to achieve a desired goal. For instance, in Ivory Coast, the civil unrest following the disputed presidential election of 2010 disrupted healthcare service provision. The local health system achieved resilience by adopting a number of collateral pathways. For instance, non-physicians were allowed to prescribe medicines, and medicines were sourced externally through the United Nations (UN) system.^28^ In the United States, one of the strategies employed to improve the resilience of the health safety net programme, in the face of federal budget cuts, was to increase focus on insured patients in order to generate revenues that help cross-subsidize uncompensated care.^21^ Collateral pathways enhance resilience by providing for alternative courses of action; when a system experiences disruption or challenges on one pathway, an alternative pathway is utilized to achieve the same goal. This characteristic draws from the feature of systems as complex adaptive systems (CAS).^51^ Related to the notion of collateral pathways is redundancy. Redundancy is the inclusion of extra components or resources that are not strictly necessary to functioning, in case of failure in other components or resources. Sheffi and Rice^41^ examined the resilience of the transport sector to supply chain disruptions caused by acute manmade and natural shocks and found that organizations were more resilient when they kept additional resources in reserve (over and above the required levels) to be used in case of an emergency.

#### Governance Process

Governance practices are also shown to influence the resilience of organizations to both acute and everyday challenges, in both health and other sectors.^15,18,31,36,44^ Governance is used here to mean the rules and processes that guide operations and affairs of organizations.^52^ A number of governance practices are identified as critical for organizational resilience. The first is decentralization; resilient organizations adopted a form of governance characterized by distributed control, rather than top down hierarchy, under central control.^15,18,36,44^ This allowed systems to be more responsive to changes in the environment by empowering local actors and provided the necessary flexibility that facilitated timely responses to everyday challenges and in times of crisis.^15,18,36,44^ For example, when the healthcare system in Ivory Coast was disrupted by civil war, the fact that drug management and distribution had been decentralized from the federal to the state level made it much easier to transport drugs to and from the local drug store whenever transport routes were secure.^15^ This allowed for reduced disruption of drug supply during the civil war. A shift from a centralized and top-down decision making system, to a decentralized system with bottom-up decision making that was characterized by local and regional initiatives was shown to contribute to the resilience of the California water management system to chronic economic, political, and environmental challenges in the United States of America.^18^ Another governance practice that distinguished resilient from non-resilient organizations was non-linear planning. For example, the federal and state laws required the California program of water management in the United States to plan in a linear, stepwise fashion; defining problems, identifying possible interventions, and refining them into implementable actions. This approach was linear in the sense that it did not allow for feedback loops between different stages (eg, redefining problems based on deliberations on agreeable interventions) or the simultaneous considerations of problems and solutions. This approach was however found to compromise the resilience of the water management system to everyday environmental, economic, and political challenges, and was instead replaced by a non-linear approach that was evolving, open-ended, iterative, and characterized by feedback loops between stages, and learning by trial and error.^18^ Non-linear planning is compatible with CAS which are typically characterized by non-linear dynamics. Resilient organizations also practiced deliberative democracy, rather than representative democracy.^18,23,29,31,44^ Deliberative democracy differs from representative democracy in that deliberation, not mere voting, is the basis of decision making. Decision making by deliberative democratic principles empowered actors and built trust, motivation and commitment.^18,23,44^ Related to deliberative democracy, organizations that embraced transparency in their processes and decisions, especially during turbulent times were found to be more resilient.^24^ Another governance feature that influences the resilience of organizations to both acute and everyday challenges is the degree of coordination between different functions and parts of the organization.^18,31,33^ McKenzie et al^31^ examined the resilience of the Nigerian health system to a disease outbreak such as Ebola, and found that the fragmentation of the health sector, characterized by lack of coordination between the delivery of services, management of human resources, and health financing, was the most significant threat to resilience because it resulted in duplication of efforts, wastage and impaired coordination during crisis. McManus et al^33^ found that organizations (10 case study organizations drawn from varied sectors) whose functions and parts operated in an uncoordinated and “silo” fashion where less resilient to manmade and natural disasters, compared to organizations that had coordinated systems. Integrating delivery systems enhances coordination, effectiveness and efficiency as well as eliminating constraints, managerial uncertainty and wastage of resources.^31,33^

#### Leadership Practices

The importance of leadership practices to the resilience of organizations is a recurrent theme across the selected papers in both the health and other sectors, and for both acute, and everyday challenges.^17,21-23,26,29,32,36,39,43,45^ For example, Seville et al^42^ examined the resilience of road infrastructure organizations to earthquakes in New Zealand and found that while some organizations had comprehensive risk management and business continuity plans, resilience depended not only on how well these plans were applied, but also on the leadership capacity of the organizations. In South Africa, health facilities that had dedicated leaders were found to be more resilient to everyday challenges (eg, chronic staff shortages, and resource scarcity) compared to health facilities whose leaders were not dedicated.^29^ One of the important roles of leaders was creating a clear and shared vision.^18,26,33^ A shared vision provided a point of focus and stimulated agency among staff during challenges and crises. McManus et al^32^ found that leadership practices characterized by visibility, and availability contributed to the resilience of organizations to acute natural and manmade shocks in New Zealand. Leadership in resilient organizations was characterized by inclusive decision making.^18,29,33,39^ Leaders ensured that relevant stakeholders were included and contributed to decision making. This nurtured the resilience of organizations to both everyday challenges, and acute shocks by building trust, empowering, motivating and creating commitment among staff and other stakeholders. For example, Pal et al^39^ examined the resilience of textile firms in Sweden to economic crisis and found that firms that had transparent and inclusive leaders were more resilient compared to those that had less transparent, non-inclusive leaders. A distinction was also made between leadership practices that were not aligned to the complex adaptive nature of systems, and those that were aligned. Booher and Innes^18^ found that the resilience of water management organizations to everyday environmental, economic and political challenges was improved when managers exercised complex leadership: rather than being controlling and directive, the leaders were mediators and facilitators of the actions of organization actors, and influenced conditions to guide interactions.

#### Organizational Culture

Two cultural practices are identified as key to organizational resilience. First is the organizations attitude towards everyday and acute challenges.^22,45^ The ability of leaders and other staff to view challenges from an opportunistic perspective is important for resilience.^22,33,45^ Resilient organizations consider challenges as learning opportunities, and used these experiences to develop capabilities that improve their resilience.^33,40,45^ For example, Oluwasoye and Ugonna^37^ found that the resilience of multinational oil corporations to environmental disasters (eg, gas flaring and oil spills) in Nigeria was weakened by a tendency towards denial of problems and potential risks. They observe that improving the organisational resilience of these organizations will entail, among others, the willingness for organization’s leaders to own the problems and seek to learn from the experiences.^37^ Sawalha^22^ found that an organizational culture characterised by lack of organisational learning from past experiences weakened the resilience of insurance companies to everyday challenges (competition, loss of customers, financial losses, political instability) in Jordan. Second, resilient organizations support creativity and innovation.^30,44^ Mafabi et al^30^ examined the resilience of 51 public corporations in Uganda to unspecified acute shocks and observed that when organizations have a creative climate, staff are motivated to generate new ideas, which strengthen organisational resilience. Staff in organizations with a poor creative climate were guarded and closed, and reluctant to offer innovative and creative ideas because they would be disregarded. A creative climate is thought to be imperative for providing a conducive environment for organizational adaptation and transformation in the face of challenges.^30,44^ Resilient organizations nurtured creativity by providing time and resources for experimentation, rewarded innovation, tolerance for failure, and an atmosphere in which employees felt safe to share new ideas.^30,44^

#### Human Capital

All the selected papers recognize the important role that human resources play in the resilience of organizations to everyday challenges and acute shocks. Having an adequate number of human resources and the requisite skills was highlighted as a critical contributor to resilience. However, beyond numbers and skills, ensuring that staff are adequately motivated and fully committed to organizational goals was highlighted as more important.^15,21,23,28,45^ For instance, Ager et al^15^ found that the resilience of the healthcare system in Yobe state, Nigeria, in the face of Boko Haram terrorism insurgency was enhanced by staff commitment and motivation, that was characterized by acceptance of challenging working shift arrangements and taking of additional responsibilities through informal task shifting. In Ivory Coast, the continuity of service delivery of the HIV program was made possible by the commitment and motivation of health workers, who continued to come to work despite delays in salaries, and security concerns.^28^ One ways of ensuring that staff are motivated and committed is prioritizing staff wellbeing.^39,45^ Walker et al^45^ found that the resilience of infrastructure organizations (air travel, banking, telecommunications, roading and water/waste services) to earthquakes in New Zealand was enhanced in those organizations where the wellbeing of staff was prioritized. This was achieved by creating a positive social environment where staff were free to express emotions and share information, providing staff with resources that were adequate to match their work demand, actively listening, monitoring, and addressing changing staff stresses, and flexibility around staff-needs.^45^ Employee engagement was reduced when managers lacked emotional intelligence. Macey and Schneider^53^ define work engagement as a fulfilling, positive, work-related state of mind that is characterized by dedication and vigor. In organizations that had a high level of employee engagement, staff dedication and commitment made them to focus on the needs of the organization despite the existence of a crisis.^45^

### Social Networks and Collaboration

How well organizations establish and leverage their networks determines the extent to which they are resilient to everyday challenges and acute shocks.^15-18,21,26,27,33,35,43^ For example, during the civil war in Ivory Coast, the resilience of the HIV service delivery program was enhanced by the relationship between healthcare facilities that allowed them to share drugs with those running out of stock.^28^ The resilience of the California water management system to everyday environmental, economic, and political challenges was enhanced by the networked nature of its agencies and a culture of collaboration among them.^18^ Social networks offer avenues for increased mobilization and transfer of knowledge, dissemination of innovations, thus increasing the overall resilience of systems.^54^ Collaboration among organizations in a networked environment also expands resources that can be drawn on, ability to learn, and its capacity to respond.^16,45^ Andrew et al^16^ examined the resilience of public (government agencies), private (small businesses and corporations) and non-governmental organizations (temples and community groups) to floods in Thailand and found that organizations that strategically collaborate with others are able to mobilize additional resources that are crucial for emergency response. The study found that organizations in urban and sub-urban areas were less resilient compared to those in rural areas because urban settings were more fragmented and hence had less social support and cohesion compared to rural ones, making them more vulnerable to disruptions.^16^

## Discussion

Drawing from our review, we make several observations that are relevant to nurturing the resilience of health systems. First, a recurrent theme across resilient literature is the recognition of systems and organizations as CAS. A CAS framework is predominantly used to understand and examine organizational resilience. This resonates with conceptual literature that views resilience as an emergent property of systems. CAS are composed of multiple interconnected components whose interaction is dynamic and non-linear.^55,56^ CAS are characterized by *self - organization and emergence.*^55,57^ Self-organization occurs when system components mutually adjust their configurations in response to environmental signals.^58^ Self-organization of the system leads to *emergence*, the appearance of unpredictable outcomes such as new structures, and patterns of behaviour.^59^ Complex interactions between system components provide multiple paths for action and enable organizations to adapt to multiple environmental changes.^57^ The attributes of resilient organizations that we identified in this literature review, including the use of collateral pathways, governance practices that promote flexibility, nurturing of social networks and collaborations neatly map onto the view of resilience as an emergent property of CAS. Further, complex leadership practices that foster productive emergence rather than prescriptive control recognize the CAS nature of systems. Leaders who recognize complexity seek to forge connections and networks among system agents because they appreciate the value of social networks to organizational resilience.^60,61^ They seek to create organizational environments that incentivize the emergence of positive adaptations, rather than prescribe solutions.^60,61^ They see the system whole rather than as isolated components.^60,61^ Given that health systems are CAS, these attributes of CAS should be recognized and nurtured to promote the resilience of health systems.

Second, empirical literature recognizes that resilience is both a function of planning for and preparing for future crisis (planned resilience), and adapting to change and disruptions (adaptive resilience). It is however recognized that planning alone is not sufficient, and that organizations must focus on developing a capacity to adapt to changing environments. While planning might help mitigate the effect of acute shocks to the health system, whether or not the health system is able to maintain core functions of delivering quality healthcare services in an efficient and equitable way also depends on how well it adjusts to the post crisis phase. Investing in structures and processes that promote the adaptive capacity of health systems is therefore important. Further, resilience to everyday challenges cannot be achieved by risk management and organizational continuity plans because such plans are often aimed at isolated events that are transient and have clear boundaries. Everyday challenges are unpredictable, multiple, and have fuzzy boundaries in the sense that they are interconnected in complex ways. To overcome these challenges, health systems will need to adapt in creative and innovative ways, and transform to new and improved forms of operations.

Third, while the empirical literature from other sectors has embraced not only the notion of resilience to acute shocks, but also resilience to chronic or everyday challenges, it appears that the health sector is largely focused on acute shocks. This is perhaps understandable given that the resilience debate in the health sector has been inspired by the occurrence of acute shocks, most notably the 2014-2016 Ebola outbreak. However, general experience of health systems, especially in low- and middle-income countries, highlights the fact that health systems also face chronic everyday challenges such as dysfunctional policies, chronic underfunding, limited human resource capacity, and high levels of disease burden.^62^ In the same way that organizations outside the health sector recognize and strategize on how to nurture resilience to chronic, everyday challenges, health systems also need to focus on nurturing what Gilson et al^62^ call “everyday resilience.” This is crucial not only because everyday resilience has an inherent value, but also because it has an instrumental value in promoting the resilience of organizations to acute shocks.^33^ This can be explained by the finding from our review that everyday resilience, and resilience to acute shocks share attributes.

A fourth lesson to draw from this literature review is that organizational software is at least, if not more, important than organisational hardware, in nurturing health system resilience. While the hardware of material resources are key ingredients for health system resilience, soft aspects of the system such as adequate planning, governance practices, leadership practices, organizational culture, staff motivation and commitment are much more important in and of themselves, and also in ensuring that the hardware is adequately mobilized for resilience. For instance, our review found that social networks and collaboration (system software) were crucial in mobilizing material resources (system hardware) that was necessary for resilience.

## Conclusion

To our knowledge, this is the first review of empirical studies that focuses on organizational resilience, and includes literature from the health sector. Understanding the attributes of resilient systems and strategies that can be employed to nurture resilience will be useful in informing global health efforts to strengthen health systems. Further studies should focus on testing the attributes identified by this review in the health sector, as well as identifying other factors that characterize resilient health systems. For the concept to offer insights that are useful in improving health systems performance, it is imperative that sufficient evidence about how organizations and systems experience, and deal with, both chronic stresses and acute shocks is generated. The concepts and principles that have dominated much of resilience literature will need to be tested in the real world of health systems. In such work, frameworks should be developed that focus not only on resilience to sudden shocks, but also resilience to everyday challenges. Moreover, methods that appreciate the CAS nature of systems, such as system dynamic modeling, should be applied and explored.

## Acknowledgements

Edwine Barasa, Rahab Mbau, and Lucy Gilson are members of the Consortium for Resilient and Responsive Health Systems (RESYST). This document is an output from a project funded by the UK Aid from the UK Department for International Development (DFID) for the benefit of developing countries. However, the views expressed and information contained in it are not necessarily those of or endorsed by DFID, which can accept no responsibility for such views or information or for any reliance placed on them. Edwine W. Barasa is supported by a Wellcome Trust Training Fellowship (#107527). The funders had no role in the writing of this paper or in the decision to submit for publication. This work is published with the permission of the Director of KEMRI.

## Ethical issues

Not applicable.

## Competing interests

Authors declare that they have no competing interests.

## Authors’ contributions

EB and LG conceptualized the review; EB and RM conducted the literature search and selection. EB and RM conducted the data extraction. EB conducted the analysis, and synthesis. EB developed the first draft of the paper. All authors contributed to subsequent and final drafts.

## Authors’ affiliations

^1^Health Economics Research Unit, KEMRI Wellcome Trust Research Programme, Nairobi, Kenya. ^2^Nuffield Department of Medicine, University of Oxford, Oxford, UK. ^3^School of Public Health and Family Medicine, University of Cape Town, Cape Town, South Africa. ^4^Department of Global Health and Development, London School of Hygiene and Tropical Medicine, London, UK.

## References

[1] Thomas S, Keegan C, Barry S, Layte R, Jowett M, Normand C (2013). A framework for assessing health system resilience in an economic crisis: Ireland as a test case. BMC Health Serv Res.

[2] Kruk ME, Myers M, Varpilah ST, Dahn BT (2015). What is a resilient health system? Lessons from Ebola. Lancet.

[3] Kruk ME, Ling EJ, Bitton A (2017). Building resilient health systems: a proposal for a resilience index. BMJ.

[4] Haldane V, Ong SE, Chuah FL, Legido-Quigley H (2017). Health systems resilience: meaningful construct or catchphrase?. Lancet.

[5] Kieny MP, Evans DB, Schmets G, Kadandale S (2014). Health-system resilience: reflections on the Ebola crisis in western Africa. Bull World Health Organ.

[6] Blanchet K, Nam SL, Ramalingam B, Pozo-Martin F (2017). Governance and Capacity to Manage Resilience of Health Systems: Towards a New Conceptual Framework. Int J Health Policy Manag.

[7] Norris FH, Stevens SP, Pfefferbaum B, Wyche KF, Pfefferbaum RL (2008). Community resilience as a metaphor, theory, set of capacities, and strategy for disaster readiness. Am J Community Psychol.

[8] Holling CS (1973). Resilience and stability of ecological systems. Annu Rev Ecol Syst.

[9] Folke C (2006). Resilience: The emergence of a perspective for social–ecological systems analyses. Glob Environ Change.

[10] Pickett STA, Cadenasso ML, Grove JM (2004). Resilient cities: meaning, models, and metaphor for integrating the ecological, socio-economic, and planning realms. Landsc Urban Plan.

[11] Pike A, Dawley S, Tomaney J (2010). Resilience, adaptation and adaptability. Cambridge Journal Regions Economy Society.

[12] Bhamra R, Dani S, Burnard K (2011). Resilience: the concept, a literature review and future directions. Int J Prod Res.

[13] Vogus TJ, Sutcliffe KM. Organizational resilience: towards a theory and research agenda. Paper presented at: ISIC. IEEE International Conference; 2007.

[14] Achour N, Price AD (2010). Resilience strategies of healthcare facilities: present and future. International Journal of Disaster Resilience in the Built Environment.

[15] Ager AK, Lembani M, Mohammed A (2015). Health service resilience in Yobe state, Nigeria in the context of the Boko Haram insurgency: a systems dynamics analysis using group model building. Confl Health.

[16] Andrew S, Arlikatti S, Siebeneck L, Pongponrat K, Jaikampan K (2016). Sources of organisational resiliency during the Thailand floods of 2011: a test of the bonding and bridging hypotheses. Disasters.

[17] Beermann M (2011). Linking corporate climate adaptation strategies with resilience thinking. J Clean Prod.

[18] Booher DE, Innes JE (2010). Governance for Resilience : CALFED as a Complex Adaptive Network for Resource Management. Ecol Soc.

[19] Burke S, Thomas S, Barry S, Keegan C. A Working Paper from the Resilience project in the Centre for Health Policy and Management, School of Medicine, Trinity College Dublin. https://www.tcd.ie/medicine/health_policy_management/assets/pdf/Resilience-working-paper-March-2014.pdf. 2014.

[20] Christopher M, Peck H (2004). Building the Resilient Supply Chain. The International Journal of Logistics Management.

[21] Felland LE, Lesser CS, Staiti AB, Katz A, Lichiello P (2003). The resilience of the health care safety net, 1996-2001. Health Serv Res.

[22] Sawalha IH (2015). Managing adversity: understanding some dimensions of organizational resilience. Management Research Review.

[23] Hassall ME, Sanderson PM, Cameron IT. Industry Perspectives on Organisational Resilience. RISK conference; Brisbane, Australia; May 2014:28-30.

[24] Herrfahrdt-Paehle E, Pahl-Wostl C (2012). Continuity and Change in Social-ecological Systems: the Role of Institutional Resilience. Ecol Soc.

[25] Heese M, Kallus W, Kolodej C. Assessing Behaviour towards Organizational Resilience in Aviation. 5th REA Symposium managing trade offs; 24th-27th June 2013; Soesterberg, Netherlands; 2014:67-74.

[26] Kachali H, Stevenson JR, Whitman Z, Seville E, Vargo J, Wilson T (2012). Organisational Resilience and Recovery for Canterbury Organisations after the 4 September 2010 Earthquake. Australasian Journal of Disaster and Trauma Studies.

[27] Lapao LV, Silva A, Pereira N, Vasconcelos P, Conceicao C (2015). Ebola impact on African health systems entails a quest for more international and local resilience: the case of African Portuguese speaking countries. Pan Afr Med J.

[28] Lembani M, Mohammed A, Abdulwahab A, et al. A Case Study of Technical Assistance to HIV Services in Cote d’Ivoire in the Context of Civil Unrest Following the Disputed Presidential Election of 2010. Cape Town; 2014.

[29] Lembani M, Mohammed A, Abdulwahab A, et al. A Case Study of Maternal Health Service Provision in OR Tambo District, Eastern Cape, in the Context of Chronic Poor Health Performance. Cape Town; 2015.

[30] Mafabi S, Munene JC, Ahiauzu A (2013). Organisational Resilience: Testing the Interaction Effect of Knowledge Management and Creative Climate. Journal of Organizational Psychology.

[31] McKenzie A, Abdulwahab A, Sokpo E, et al. Building a Resilient Health System: Lessons from Northern Nigeria. IDS Working Paper; Volume 2015 No. 454.

[32] McManus S, Seville E, Brunsdon D, Vargo J. Resilience Management: A Framework for Assessing and Improving the Resilience of Organisations. Resilient Organisations Research Report; 2007:79-79.

[33] McManus S, Seville E, Vargo J, Brunsdon D (2008). Facilitated Process for Improving Organizational Resilience. Nat Hazards Rev.

[34] Nilakant V, Walker B, Rochford K, Van Heugten K (2013). Leading in a Post-disaster Setting: Guidance for Human Resource Practitioners. New Zealand Journal Employment of Relations.

[35] Nyikuri M, Tsofa B, Barasa E, Okoth P, Molyneux S (2015). Crises and Resilience at the Frontline-Public Health Facility Managers under Devolution in a Sub-County on the Kenyan Coast. PLoS One.

[36] Olsson P, Folke C, Berkes F (2004). Adaptive comanagement for building resilience in social-ecological systems. Environ Manage.

[37] Oluwasoye M, Ugonna N (2015). Environmental risk: exploring organisational resilience and robustness. Int J Sci Eng Res.

[38] Andersson R, Rudrajeet P, Torstensson H (2012). Organisational resilience through crisis strategic planning: a study of Swedish textile SMEs in financial crises of 2007-2011. International Journal of Decision Sciences, Risk and Management.

[39] Pal R, Torstensson H, Mattila H (2014). Antecedents of organizational resilience in economic crises—an empirical study of Swedish textile and clothing SMEs. Int J Prod Econ.

[40] Sandanda M (2014). What determines the resilience of retail business in an unstable business environment? Evidence from Harare Province. University of Zimbabwe Business Review.

[41] Sheffi Y, Rice JB Jr (2005). A Supply Chain View of the Resilient Enterprise. MIT Sloan Management Review.

[42] Seville E, Brunsdon D, Dantas A, Le Masurier J, Wilkinson S, Vargo J. Building Organisational Resilience: A New Zealand Approach. http://hdl.handle.net/10092/649. Published 2006. 21339112

[43] Seville E, Brunsdon D, Dantas A, Le Masurier J, Wilkinson S, Vargo J (2008). Organisational resilience: Researching the reality of New Zealand organisations. J Bus Contin Emer Plan.

[44] Stephenson A, Seville E, Vargo J, Roger D. Benchmark Resilience: A study of the resilience of organizations in the Auckland region. Auckland: Resilient Organizations Research Group (ResOrgs); 2010:1-49.

[45] Walker B, Nilakant V, Baird R. Promoting Organisational Resilience through Sustaining Engagement in a Disruptive Environment: What are the implications for HRM ? Research Forum. 2014:1-20.

[46] Zhong S, Hou XY, Clark M (2014). Disaster resilience in tertiary hospitals: a cross-sectional survey in Shandong Province, China. BMC Health Serv Res.

[47] CASP UK. Critical Appraisal Skills Programme (CASP) Check lists. http://www.casp-uk.net/casp-tools-checklists. Accessed July 31, 2017. Published 2017.

[48] Hannes K. Critical appraisal of qualitative research. In: Noyes J, Booth A, Hannes K, et al, eds. Supplementary Guidance for Inclusion of Qualitative Research in Cochrane Systematic Reviews of Interventions. Cochrane Collaboration Qualitative Methods Group; 2011. http://cqrmg.cochrane.org/supplemental-handbook-guidance. Accessed September 1, 2015.

[49] Braun V, Clarke V (2006). Using thematic analysis in psychology. Qual Res Psychol.

[50] Bristow G, Healy A (2014). Building Resilient Regions: Complex Adaptive Systems and the Role of Policy Intervention. Raumforsch Raumordn.

[51] Marion R. The Edge of Organization: Chaos and Complexity Theories of Formal Social Systems. London: Sage Publications; 1999.

[52] Brinkerhoff DW, Bossert TJ (2014). Health governance: principal-agent linkages and health system strengthening. Health Policy Plan.

[53] Macey WH, Schneider B (2008). The meaning of employee engagement. Ind Organ Psychol.

[54] Moore ML, Westley F (2011). Surmountable chasms: networks and social innovation for resilient systems. Ecol Soc.

[55] Begun JW, Zimmerman B, Dooley K. Health Care Organizations as Complex Adaptive Systems. In: Mick SM, Wyttenbach M. San Francisco: Jossey-Bass; 2003:253-288.

[56] Complex adaptive systems. London: Health Foundation; 2010.

[57] Marion R, Bacon J (2000). Organizational Extinction and Complex Systems. Emergence.

[58] Lindberg C, Nash S, Lindberg C. On the edge: Nursing in the age of complexity. Bordentown: Plexus Press; 2008.

[59] Zimmerman B. The Sage handbook of complexity and management. Thousand Oaks, CA: Sage Publishers; 2010.

[60] Marion R, Uhl-Bien M (2001). Leadership in Complex Organizations. The Leadership Quartely.

[61] Ford R (2009). Complex leadership competency in health care: towards framing a theory of practice. Health Serv Manage Res.

[62] Gilson L, Barasa E, Nxumalo N (2017). Everyday resilience in district health systems: emerging insights from the front lines in Kenya and South Africa. BMJ Glob Health.

